# Oral Ulcerations

**DOI:** 10.5811/westjem.2015.8.28358

**Published:** 2015-12-08

**Authors:** Brandon Fetterolf, Alexandra Zaballa, Jared Strote

**Affiliations:** * Madigan Army Medical Center, Department of Emergency Medicine, Tacoma, Washington; † University of Washington School of Medicine, Seattle, Washington; ‡ University of Washington, Division of Emergency Medicine, Seattle, Washington

A 35-year-old male presented with lower gum pain associated with fever, chills, and sore throat. His medical history included intravenous drug use, human immunodeficiency virus infection, and hepatitis C. Physical exam revealed tachycardia, a temperature of 38.9°C, anterior cervical lymphadenopathy, halitosis, an edematous lower lip, and purulent ulcers anterior and posterior to lower central incisors with marked tenderness and erythema (Figure). His laboratory work was notable for a low white blood cell count (2.6 thousand/μl), neutropenia (0.11 thousand/μl), a low absolute CD4 lymphocyte count (0.5 thousand/μl), and elevated C-reactive protein (129mg/L) and sedimentation rate (23mm/hr). A computed tomography study showed a 0.5×1.3×0.3cm abscess anterior to the mandibular symphysis.

## DIAGNOSIS

### Acute Necrotizing Ulcerative Gingivitis

Intravenous vancomycin, piperacillin/tazobactam, and fluconazole were initiated. Oral-maxillofacial surgery was consulted and performed debridement. Continued intravenous antibiotics and chlorhexidine rinses were recommended.

The patient was admitted to the medicine service for three days. Herpes simplex and syphilis studies, sent to rule out other causes of the patient’s ulcers, were negative. His oral infection improved and his absolute neutrophil count, ultimately thought to be low due to acute infection, normalized. He was discharged with outpatient oral antimicrobial therapy and was ultimately lost to follow up.

Necrotizing periodontal disease presents as interdental necrosis with “punched out” ulcerative papilla, gingival bleeding, and pain. It usually affects young adults, commonly military and college students.^1^,^2^ Secondary features of disease include foul-smelling breath, yellowish-white or grayish “pseudomembrane,” lymphadenopathy, and fever.^1^ Risk factors include immunosuppression, psychological stress, smoking, poor oral hygiene, and poor nutrition.^1^ Common organisms include Bacteriodes, Prevotella, Fusebacterium, and Selenomonas but fungal infections also occur.^2^ Treatment usually involves debridement, oral antimicrobial rinses, and antibiotics for any signs of systemic involvement.^1^

## Figures and Tables

**Figure:**
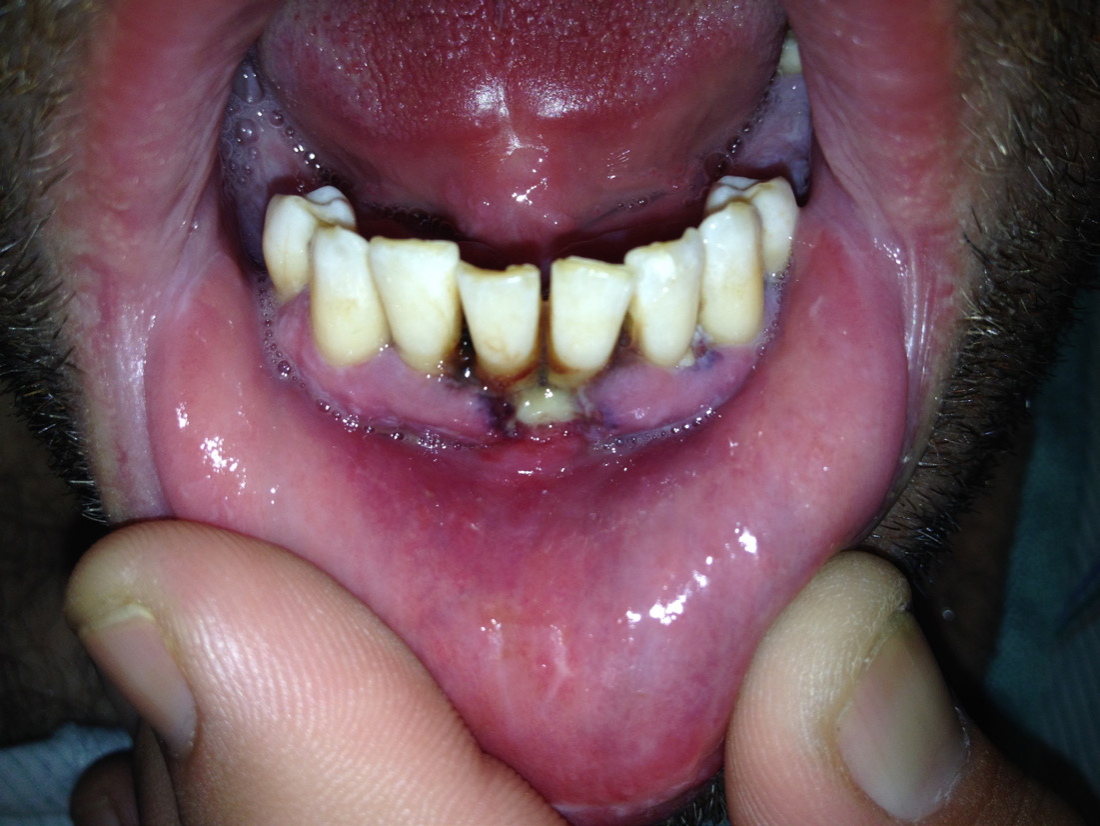
Lower lip and gums with inflammation and ulceration.
